# One-pot conversion of disaccharide into 5-hydroxymethylfurfural catalyzed by imidazole ionic liquid

**DOI:** 10.1038/srep26067

**Published:** 2016-05-16

**Authors:** Yongshui Qu, Li Li, Quanyuan Wei, Chongpin Huang, Piotr Oleskowicz-Popiel, Jian Xu

**Affiliations:** 1 State Key Laboratory of Biochemical Engineering, Institute of Process Engineering, Chinese Academy of Sciences, Beijing, 100190, China; 2 Beijing University of Chemical Technology, Beijing, 100029, China; 3 Beijing Municipal Research Academy of Environmental Protection, Beijing, 100037, China; 4 Institute of Environmental Engineering, Faculty of Civil and Environmental Engineering, Poznan University of Technology, Berdychowo 4, 60-965, Poznan, Poland

## Abstract

Conversion of carbohydrate into 5-hydroxymethylfurfural (5- HMF), a versatile, key renewable platform compound is regarded as an important transformation in biomass-derived carbohydrate chemistry. A variety of ILs, not only acidic but also alkaline ILs, were synthesized and used as catalyst in the production of 5-HMF from disaccharide. Several factors including reaction temperature, IL dosage, solvent and reaction time,were found to influence the yield of 5-HMF from cellobiose. Of the ILs tested, hydroxy-functionalized ionic liquid (IL), 1-hydroxyethyl-3-methylimidazolium tetrafluoroborate ([AEMIM]BF_4_) showed the highest catalytic activity and selectivity. 5-HMF yield of 68.71% from sucrose was obtained after 6 hrs at 160 °C. At the same condition with cellobiose as substrate, 5-HMF yield was 24.73%. In addition, 5-HMF also exhibited good stablity in this reaction system. Moreover, a kinetic analysis was carried out in both acidic and alkaline IL-catalyzed system, suggesting main side reaction in the conversion of fructose catalyzed by acidic and alkaline IL was polymerization of fructose and 5-HMF degradation, respectively.

The massive consumption of fossil resources has made it an essential task to find new feedstocks. Biomass has a remarkable potential to serve as an alternative of fossil resources for the production of energy and organic compounds^1^,^2^,^3^. 5-hydroxymethylfurfural (5- HMF) has been reported to be one of the top building block chemicals obtained from biomass^4^,^5^. A broad range of value added compounds can be synthesized from it, such as 1,6-hexanediol^6^, 5-hydroxymethyl-2-furancarboxylic acid (HMFCA)^7^, 5-ethoxymethylfurfural (EMF)^8^, 2,5-dihydroxymethylfurfural (DHMF) and 5-hydroxymethyl furanoic acid (HMFA)^9^, 2,5-furandicarboxylic acid^10^, 2,5-diformylfuran (2,5-DFF)^11^, 5-formyl-2-furancarboxylic acid (FFCA) and 2,5-furandicarboxylic acid (FDCA)^12^.

Since the emergence of room temperature ionic liquids (ILs), it has been playing an important role in various aspects of chemical researches. The distinctive properties give ILs a chance to be used extensively in catalysis^13^,^14^ and subsequently in biomass processing^15^,^16^. In marked contrast to many studies with the application of ILs as solvent or co-solvent on 5-HMF production^5^,^17^, very few have been focused on ILs’ catalytic activity. Although there is a growing literature on dehydration of C-6 carbohydrates, mainly fructose and glucose^18^,^19^, for the production of 5-HMF, little is available on conversion of dissacharide. When 1-methyl-3-octylimidazolium chloride was used as solvent and CrCl_2_/HCl as catalyst, 5-HMF yield could reach 82 ± 3.7% from sucrose, higher than that with ZnCl_2_/HCl as catalyst which was 58 ± 2.7%^20^. Ilgen^21^ demonstrated the production of 5-HMF in ILs with the presence of CrCl_2_ and a maximum 5-HMF yield of 42% was obtained from the dehydration of sucrose under 100 °C for 1 h. Lima *et al*.^22^ reported a 5-HMF yield of 58% achieved using [BMIM]Cl as reaction medium with a mass ratio of IL to sucrose at 10 with CrCl_3_·H_2_O at 100 °C for 4h. The conversion of sucrose and cellobiose to 5-HMF catalyzed by Lewis acid (SnCl_4_) was studied by Hu *et al*.^23^, using 1-ethyl-3-methylimidazolium tetrafluoroborate ([EMIM]BF_4_) as solvent at 100 °C for 3 h and obtained the highest yield of 57% and 65%, respectively. All the above-mentioned ILs are neutral or acidic, and dehydration is promoted due to enhanced dissolution of catalysts and substrates. Although alkaline ILs, display high activity for some Brønsted base-catalyzed reactions (Knoevenagal condensation reaction and Michael addition^24^,^25^) and also can be switched repeatedly by bubbling CO_X_ and NO_X_ through the solution alternately^26^,^27^,^28^,^29^, to our knowledge there have been little reports of the use of alkaline ILs as a catalyst for the conversion of carbohydrates to 5-HMF.

In the present study, Brønsted acidic IL, 1-(3-sulfonic acid) propyl-3-methylimidazolium phosphotungstate ([MIMPS]_3_PW_12_O_40_), and a variety of alkaline ILs, 1-butyl-3-methylimidazolium hydroxide ([BMIM]OH), 1-ethyl-3-methylimidazolium hydroxide ([EMIM]OH), 1-butyl-3-methylimidazolium carbonate ([BMIM]HCO_3_) and hydroxy-functionalized IL, 1-hydroxyethyl-3-methylimidazolium tetrafluoroborate ([AEMIM]BF_4_) were synthesized and used as catalyst in the conversion of disacharide (sucrose and cellobiose) to 5-HMF, which has not been reported. In addition, kinetic analysis on reaction catalyzed by different ILs was carried out to identify the catalytic effect of different ILs-catalyst on the conversion of dissacharide to 5-HMF.

## Results

### Sucrose conversion with different IL catalysts

The effect of ILs with different cations and anions, employed as catalysts for the transformation of sucrose was shown in Table 1 (P < 0.05). Among different kinds of solvents used for the conversion of sucrose with [MIMPS]_3_PW_12_O_40_ as catalyst (P < 0.05) (Table 1, entry 1–4), a comparable high yield of 5-HMF was obtained using 2-Butanol as solvent (Table 1, entry 1), compared to DMAc, NMP and DMSO (Table 1, entry 2–4), consistent with the results in a previous study^30^. The 5-HMF yield, catalyzed by alkaline ILs (Table 1, entry 5–8) was invariably higher than that obtained with the acidic catalyst (Table 1, entry 1–4). The highest yield was found to be 68.71% catalyzed by [AEMIM]BF_4_ in DMSO (Table 1, entry 8), suggesting that weak alkaline condition favored the reaction. Moreover, the yield sustained growth during the reaction time (Table 1, entry 8–10) demonstrating that 5-HMF exhibited good stability in this reaction system (P < 0.05).

### Effect of composition of the reaction medium on cellobiose conversion

**Figure 1:**
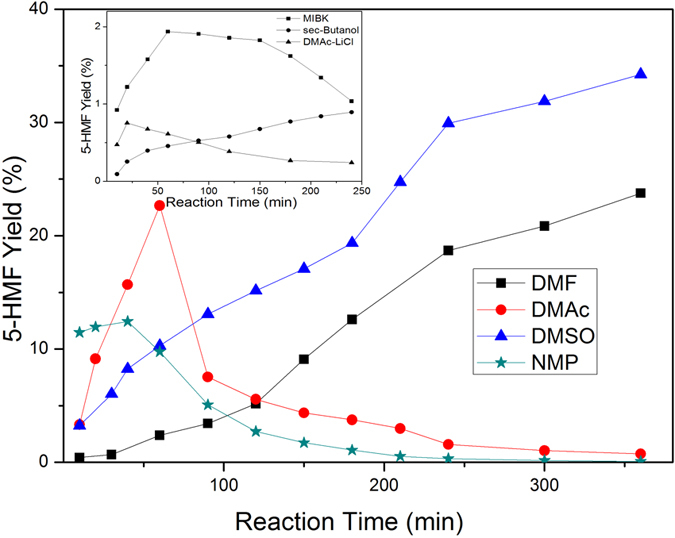
Effect of solvents on 5-HMF yield. Conditions: 0.25 g [AEMIM]BF_4_, 0.5 g cellobiose, 60 mL solvent, at 160 °C, with the presence of N_2_.

As shown in Fig. 1, there was a huge difference in the conversion of cellobiose into 5-HMF with different solvents (P < 0.01). The highest yield of 5-HMF in the reaction carried out at 160 °C in DMSO reached 34.25% in 6hrs. However, the yield was only 23.77% under the same condition with DMF as solvent.

For the reaction with DMAc and NMP as solvent, the highest yield of 5-HMF (26.29% and 12.44%, respectively) was obtained in 60 mins and 40 mins, followed by a quick reduction. In another 3 solvents, MIBK, 2-butanol and DMAc-LiCl, trace amounts of 5-HMF can be ignored. It might be concluded that 5-HMF stability is dependent on reaction solvent, *i.e.,*DMSO > DMF > DMAc > NMP > MIBK > 2-butanol > DMAc-LiCl. Moreover, the huge difference between DMAc-LiCl and DMAc indicates that the addition of metal chloride was not conducive to 5-HMF production, which was different from the reported system with ILs as solvent catalyzed by metal chloride^31^,^32^.

### Effect of temperature and reaction time on cellobiose conversion

**Figure 2:**
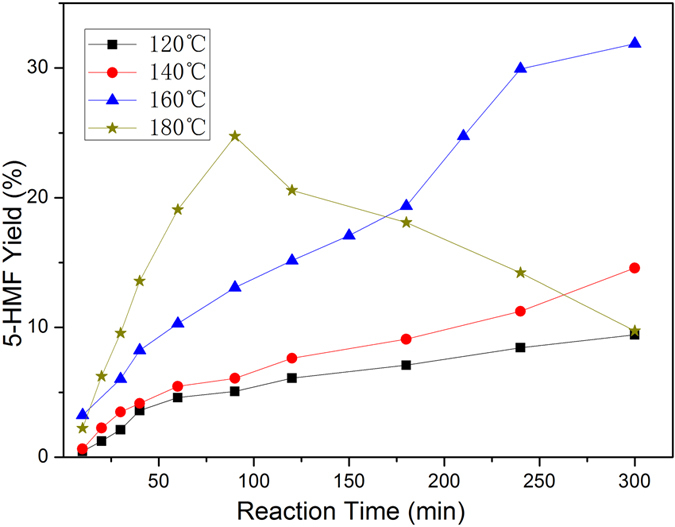
Effect of temperature and time on 5-HMF yield. Conditions: [AEMIM]BF_4_ 0.25 g, cellobiose 0.5g, in 60 mL DMSO, under N_2_.

Figure 2 shows the effect of temperature and reaction time on the conversion of cellobiose catalyzed by [AEMIM]BF_4_, using DMSO as solvent (P < 0.05). At 120 °C and 140 °C, the yields of 5-HMF increased slowly to 9.44% and 14.86% respectively after 300 mins. It presented the similar trend at 160 °C. However, the 5-HMF formation was obviously enhanced. At 180 °C, although the 5-HMF yield increased rapidly at the initial stage and the maximum 5-HMF yield of 24.73% was obtained at 90 min, it then dropped dramatically. It could thus be concluded that higher temperature could accelerate the conversion of cellobiose into 5-HMF and in the mean time, it raised the risk of 5-HMF degradation.

### Effect of the dosage of IL catalyst on the yield of 5-HMF

**Figure 3:**
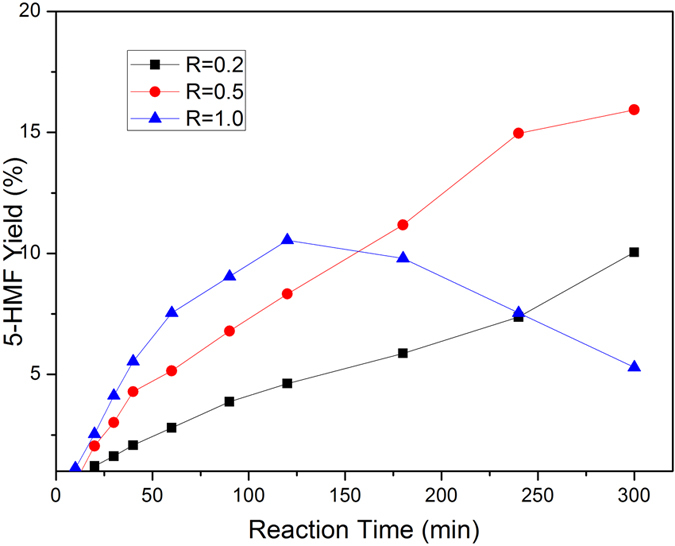
Effect of catalyst amount on 5-HMF yield. Conditions: cellobiose 0.5g, 60 mL DMSO, 160 °C, under N_2_.

Figure 3 shows the effect of the amounts of [AEMIM]BF_4_ on 5-HMF yield in the cellobiose transformation reaction (P < 0.05). An evident conclusion could be drawn from Fig. 3 that increasing the ratio of catalyst to cellobiose (R) from 0.2 to 0.5 will promote significantly the conversion of cellobiose to 5-HMF from 31.87% to 40.19% after 5 h, respectively. Further increasing the dosage of catalysts to 1.0, although the time to obtain the maximum 5-HMF yield was reduced to within 2 hrs, it was only 21.1%, much lower than that from R at 0.2 and 0.5. Moreover, the 5-HMF yield showed a sharp decrease after 2 hrs for R at 1.0. The reactivity difference might be ascribed to be the fact that IL catalyst typically exhibit high activity not only in conversion of cellobiose, but also in the polymerization of cellobiose, glucose, 5-HMF, and other small molecule compounds during degradation process.

### Kinetic analysis of the dehydration of fructose

**Figure 4:**
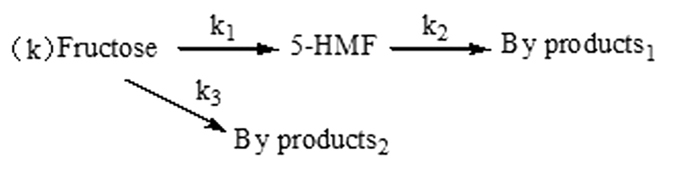
Kinetic model for dehydration of fructose catalyzed by ILs.

During conversion of disaccharide into 5-HMF, three reactions are involved, namely the hydrolysis of the disaccharide into its components (fructose and glucose), isomerization of glucose into fructose, and dehydration of fructose. In order to study the process of 5-HMF formation, the kinetic analysis of the dehydration of fructose catalyzed by ILs in DMSO was performed based on two step continuous reaction^33^ as shown in Fig. 4: hydrolysis of fructose into 5-HMF and by-products_2_; degradation of 5-HMF into by-products_1,_ including formic acid, levulinic acid, humin, and so on. These components, coming from decomposition and polymerization of 5-HMF and fructose, can be analyzed by HPLC or observation, corresponded to some publications^34^,^35^.

The conversion of 5-HMF is considered as a first-order reaction. 5-HMF generation rate and fructose consumption rate are shown as follows:


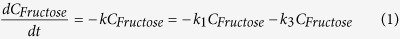



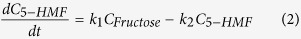


Eq. (3) and (4) can be achieved by integrating Eq. (1) and (2), *k*_1_ and *k*_2_ are step rate constants, which are constant at a definite temperature:


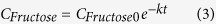



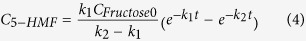


Substituting 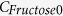 from Eq. (3) into Eq. (4) gives Eq. (5):


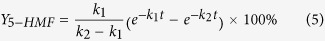


By integrating the above equation leads to the following:


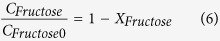



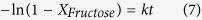


Values of 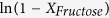 (where *X* is conversion of fructose) are plotted against reaction time (*t*) at different temperatures in order to obtain rate constants (*k*). Table 1 shows the relationship between the rate constant and reaction temperature. The results show that *k* value increases as temperature increases, meaning that the higher temperature accelerates the fructose dehydration reaction rate.

Compared to alkaline system (Table 2, entry 5–8), *k*_*3*_ in acidic system (Table 2, entry 1–4) grows faster when temperature is increased from 100 °C to 160 °C. It is why the rate of humin, coming from polymerization of fructose and 5-HMF, lactic acid, etc., rise faster at a higher temperature.


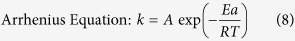



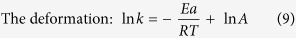


**Figure 5:**
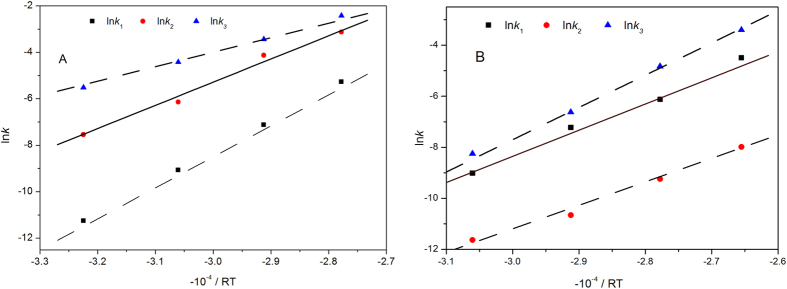
Relationship of ln*k* and −10^−4^/RT.

Figure 5A,B show relationship of ln *k* and −10^−4^/RT, using the rate constants, an Arrhenius plot is generated based on Eq. (8) & (9). The kinetic parameters for ILs catalyzed dehydration of fructose to 5-HMF, 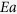
(slope) and *A* (intercept is ln *A*) are summarized in Table 3.

The values of activation energy and pre-exponential factor of 5-HMF degradation (*A*_2_ and *Ea*_2_, 1.47 × 10^7^ and 96.48 kJ·mol^−1^) catalyzed by alkaline ILs are lower than those (4.95 × 10^13^ and 136.48 kJ·mol^−1^) in acidic catalyst system, meaning that 5-HMF is degraded into small molecules such as levulinic acid, formic acid much faster than that catalyzed by [MIMPS]_3_PW_12_O_40_. Moreover, higher *Ea*_1_ and *Ea*_3_ (110.81 kJ·mol^−1^and 153.47 kJ·mol^−1^) cause the reaction rate greatly influenced by temperature, and can thus quickly reach the reaction equilibrium.

## Discussion

An efficient synthesis of 5-HMF by conversion of disaccharide has been achieved using ILs as catalysts. Several factors were found to influence the yield of 5-HMF from cellobiose, such as reaction temperature, the dosage of IL, solvent and reaction time. Of the ILs tested, [AEMIM]BF_4_ showed the highest catalytic activity and selectivity, and a 68.21% yield of 5-HMF from sucrose was obtained after 6 h when it was employed at 160 °C. At same reaction, 5-HMF yield was 24.73% in the conversion of cellobiose. In addition, a kinetic analysis was carried out on both acidic and alkaline IL-catalyzed system, suggesting main side reaction in the conversion of fructose catalyzed by acidic and alkaline IL was polymerization of fructose and 5-HMF degradation, respectively. Although the catalyzed mechanism needs to be further studied, the results obtained from the present investigation might encourage researchers to consider ILs as potential catalysts for organic reactions rather than simply as solvents.

## Methods

### Materials and experimental methods

N-methylimidazole was purchased from Aladdin Chemical Co., Ltd. 2-Chloroethylaminehydrochloride, dichloroethanol, and acetone from Shanghai Jingchun Chemical Reagent Company. Dimethylsulfoxide (DMSO), dimethyl acetamide (DMAc), N,N-dimethylformamide(DMF), n-butanol, 2-butanol, methylisobutylketone (MIBK), sodium tetrafluoroborate, ether, ethanol, sodium borate (NaBF_4_),sucrose, cellobiose, glucose and fructose were purchased from Beijing Chemical Reagent Company. All reagents were used as supplied without further purification.

The hydroxy-functionalized ILs, [AEMIM]BF_4_ 27,28,29 was synthesized as follows: 0.10 mol N-methylimidazole (8.20 g) and 0.10 mol 2-bromoethylamine hydrobromide (20.50 g) were dissolved into 60 ml ethanol, and stirred at 80 °C under nitrogen atmosphere. After 24 hrs, the mixture was filtered, and washed with ethanol for three times. The residue was dissolved into 40 ml of CH_3_CN/H_2_O (1:1, v/v) with the addition of 0.10 mol NaBF_4_ (10.98 g). It was stirred at 30 °C for another 24 hrs and then, evaporated in vacuum. The obtained yellow liquid was washed with chloroform and ether for three times, respectively. After drying at 60 °C for 24 h in vacuum, pure [AEMIM]BF_4 _was obtained with the yield of 74.4%.

Other ILs were also synthesized by two-step methods: synthesis of imidazolium chloride precursor followed by anion exchange as described by Cai *et al*.^13^. ILs containing different cations and anions, including [MIMPS]_3_PW_12_O_40 _36, [BMIM]OH^37^, [EMIM]OH^38^ and [BMIM]HCO_3 _39 were prepared.

### General procedure for the conversion of carbohydrates to 5-HMF

**Figure 6:**
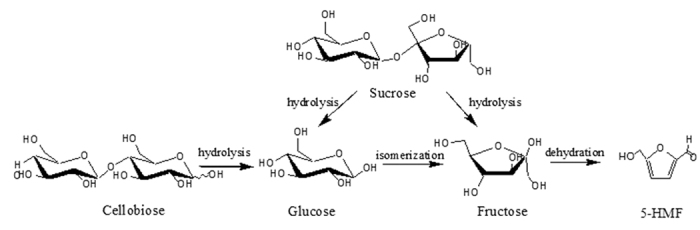
Reaction process for the dehydration.

In the dehydration reaction, 0.5 g of carbohydrate, 0.25 g of catalyst and 60 mL of solvent were mixed in a 100-mL flask equipped with a condenser and heated to certain reaction temperature. After dehydration, the mixture was analyzed by HPLC. During the dehydration reaction, disaccharide was hydrolyzed into glucose and fructose, glucose can be isomerized into fructose and the later loses three water molecules to produce HMF in the presence of catalyst (Fig. 6).

After reaction, the mixture was filtered through 0.45μm pore size membrane and the filtrate was transferred into a volumetric flask and diluted with water. 5-HMF and furfural were analyzed by HPLC (Agilent LC1260 infinity, USA) using a C18 column and UV detector at 35 °C with methanol / water (40/60, v/v) as eluent at a flow rate of 0.6 mL/min.

### Statistical Analysis

Based on data distribution, one-way and two-way analyses of variance (ANOVA) was used to compare different groups. The P value was calculated, which less than 0.05 was considered as statistically significant with P < 0.05.

## Additional Information

**How to cite this article**: Yongshui, Q. *et al*. One-pot conversion of disaccharide into 5-hydroxymethylfurfural catalyzed by imidazole ionic liquid. *Sci. Rep.* **6**, 26067; doi: 10.1038/srep26067 (2016).

## Figures and Tables

**Table 1:** Dehydration of sucrose with different ILs.

Entry	ILs	Solvent	Time (min)	5-HMF yield (%)
1	[MIMPS]_3_PW_12_O_40_	2-Butanol	180	30.29 ± 1.14
2	[MIMPS]_3_PW_12_O_40_	DMAc	300	25.92 ± 0.78
3	[MIMPS]_3_PW_12_O_40_	NMP	150	15.81 ± 0.48
4	[MIMPS]_3_PW_12_O_40_	DMSO	360	18.90 ± 0.78
5	[BMIM]HCO_3_	DMSO	180	41.17 ± 1.44
6	[EMIM]OH	DMSO	30	46.29 ± 1.22
7	[BMIM]OH	DMSO	180	54.39 ± 1.19
8	[AEMIM]BF_4_	DMSO	480	68.71 ± 0.90
9	[AEMIM]BF_4_	DMSO	30	35.46 ± 1.38
10	[AEMIM]BF_4_	DMSO	180	62.16 ± 1.41

Conditions: 0.5 g substrate, 0.25 g ILs, 60 mL solvent, 160 °C.

**Table 2:** Reaction rate constants (k) of fructose dehydration at different temperatures.

	Entry	T ( °C)	*k*(min^−1^)	*k*_*1*_(min^−1^)	*k*_*2*_(min^−1^)	*k*_*3*_(min^−1^)
A: Acidic ILs	1	100	0.00399 ± 2.83 × 10^−4^	0.00053 ± 2.83 × 10^−5^	1.3E-05 ± 2.19 × 10^−6^	0.003275 ± 4.74 × 10^−4^
	2	120	0.01205 ± 6.72 × 10^−4^	0.009665±1.34 × 10^−4^	0.000116 ± 7.78 × 10^−6^	0.00265 ± 1.56 × 10^−4^
	3	140	0.03202 ± 0.002638	0.02178 ± 3.96 × 10^−4^	0.000804 ± 6.54 × 10^−5^	0.01121 ± 8.63 × 10^−4^
	4	160	0.08802 ± 6.20 × 10^−3^	0.04375 ± 7.21 × 10^−4^	0.00515 ± 4.24 × 10^−4^	0.037116 ± 3.2 × 10^−3^
B: Alkaline ILs	5	120	0.000262 ± 7.42 × 10^−5^	0.000123 ± 4.96 × 10^−6^	8.89E-06 ± 1.91 × 10^−7^	0.000083 ± 8.49 × 10^−7^
	6	140	0.001329 ± 1.2 × 10^−5^	0.000892 ± 6.15 × 10^−5^	2.36E-05 ± 3.04 × 10^−6^	0.000408 ± 1.17 × 10^−4^
	7	160	0.008017 ± 1.46 × 10^−4^	0.00596 ± 1.7 × 10^−4^	0.000097 ± 3.25 × 10^−6^	0.001757 ± 1.08 × 10^−4^
	8	180	0.03333 ± 1.32 × 10^−4^	0.02489 ± 4.53 × 10^−4^	0.000341 ± 3.82 × 10^−5^	0.00865 ± 5.79 × 10^−4^

Conditions: 0.25 g catalyst, 0.5 g fructose, 60 mL DMSO; acidic and alkaline ILs were [MIMPS]_3_PW_12_O_40 _and [AEMIM]BF_4_.

**Table 3:** Pre-exponential factor and activation energy of fructose conversion.

	Pre-exponential factor, A (min^–1^)			Activation energy, Ea (kJ∙mol^–1^)		
	*A*_1_	*A*_2_	*A*_3_	*Ea*_1_	*Ea*_2_	*Ea*_3_
Acidic ILs	5.06 × 10^10^	4.95 × 10^13^	1.32 × 10^5^	99.78	136.48	58.84
AlkalineILs	5.44 × 10^9^	1.47 × 10^7^	9.17 × 10^14^	110.81	96.48	153.47
